# Anthelminthic treatment receipt and its predictors in Lake Victoria fishing communities, Uganda: Intervention coverage results from the LaVIISWA cluster randomised trial

**DOI:** 10.1371/journal.pntd.0008718

**Published:** 2020-10-19

**Authors:** Hellen Akurut, Richard E. Sanya, Lawrence Lubyayi, Margaret Nampijja, Moses Kizza, James Kaweesa, Robert Kizindo, Moses Sewankambo, Denis Nsubuga, Edridah Tukahebwa, Narcis B. Kabatereine, Alison M. Elliott, Emily L. Webb, for the LaVIISWA trial team

**Affiliations:** 1 Immunomodulation and Vaccines Programme, MRC/UVRI and LSHTM Uganda Research Unit Entebbe, Uganda; 2 Department of Internal Medicine, College of Health Sciences, Makerere University, Kampala, Uganda; 3 Vector Control Division, Uganda Ministry of Health, Kampala, Uganda; 4 Department of Clinical Research, London School of Hygiene and Tropical Medicine, United Kingdom; 5 MRC Tropical Epidemiology Group, London School of Hygiene and Tropical Medicine, United Kingdom; University of Cambridge, UNITED KINGDOM

## Abstract

**Background:**

Mass drug administration (MDA) is a cornerstone of control of parasitic helminths. In schistosomiasis-endemic areas with >50% of school-aged children infected, community-wide MDA with praziquantel is recommended by the World Health Organisation (WHO), with target coverage of >75%. Using data from a cluster-randomised trial of MDA treatment strategies, we aimed to describe the proportion of eligible residents who received MDA and predictors of treatment receipt, and to assess associations with helminth prevalence.

**Methods:**

In the Koome islands of Lake Victoria, Uganda, where baseline schistosomiasis prevalence (by single stool sample, Kato Katz) was 52% overall (all ages) and 67% among school-aged children, we conducted a cluster-randomised trial of community-wide, intensive MDA (quarterly single-dose praziquantel 40mg/kg; triple-dose albendazole 400mg) versus standard, Uganda government intervention (annual single-dose praziquantel 40mg/kg; 6-monthly single-dose albendazole). Twenty-six fishing villages were randomised, 13 per trial arm, for four years. At each treatment round, praziquantel treatment and the first dose of albendazole treatment were directly observed by the study team, registers of village residents were updated and the proportion receiving treatment among those eligible recorded.

**Results:**

During the four-year MDA, at each treatment round an average of 13,382 people were registered in the 26 villages (7,153 and 6,229 in standard and intensive intervention villages, respectively). Overall, the proportion of those eligible receiving praziquantel was lower than for albendazole (60% versus 65%), particularly in the standard arm (61% versus 71%) compared to the intensive arm (60% versus 62%). Albendazole receipt was lower when given concurrently with praziquantel. Absence was the commonest reason for non-receipt of treatment (81% albendazole, 77% praziquantel), followed by refusal (14% albendazole, 18% praziquantel). Proportions receiving treatment were lowest among school-aged children, but did not differ by sex. Longitudinal analysis of a subgroup of residents who did not move during the study period found that persistent non-receipt of treatment in this subgroup was rare. Refusal to receive treatment was highest among adults and more common among females.

**Conclusion:**

In schistosomiasis high-risk communities, a combination of approaches to increasing treatment coverage, such as extended periods of treatment delivery, and the provision of incentives, may be required to achieve WHO targets.

## Author summary

Mass de-worming is recommended by the World Health Organisation in communities with a heavy burden of worms, but coverage can be sub-optimal. We used data from a four-year long cluster randomised controlled trial conducted in a heavy worm burden area on Lake Victoria, Uganda, to investigate coverage of community-delivered albendazole and praziquantel treatment. We also investigated which factors were associated with receipt of treatment and with refusal. We found that, on average, a lower proportion of residents received praziquantel than albendazole, and that absence and refusal were the most common reasons for not receiving treatment. In the absence of adequate provision of water and sanitation, or of an effective vaccine, innovative approaches to increasing treatment coverage are likely to be needed to substantially reduce worm prevalence.

## Introduction

Schistosomiasis affects over 250 million people worldwide and about 90% of these are in sub-Saharan Africa [1, 2]. Left untreated, schistosomiasis can persist for many years, causing much subtle and severe morbidity. The Global Burden of Disease 2017 study estimates that schistosomiasis accounts for the loss of 1.4 million disability-adjusted life years (DALYs) [3] although this is likely to be an underestimate [4]. Mass drug administration (MDA) with praziquantel is a cornerstone of the strategy for control of schistosomiasis. In schistosomiasis-endemic “high-risk” communities with >50% of school-aged children infected, annual community-wide MDA with praziquantel is recommended by the World Health Organisation (WHO) [5], with target coverage of >75% by the year 2020 [6].

Programmatic data demonstrate that MDA causes a significant reduction in infection with *S*. *mansoni* in school-age children and this reduction may have corresponding benefits for schistosomiasis-related morbidity, such as anaemia and growth [7–9]. Although there have been many successes, studies have revealed persistently high schistosomiasis infection prevalence in some sub-Saharan African countries despite ongoing MDA efforts [10–12], and the existence of infection “hot spots” [13].

A number of studies have provided important data on MDA coverage and its determinants. At the community level, casual employment practices, lower symptom knowledge, lower risk perception, and increased experience of side effects have all been associated with lower coverage [14, 15]. At the individual and household levels, lower socio-economic status, belonging to the minority tribe, and poorer sanitation have been associated with reduced MDA coverage in Uganda, with conflicting results on whether longer term residence in the community leads to higher or lower coverage [16, 17]. Identification of groups particularly at risk of non-receipt is important for informing complementary, targeted, treatment provision strategies.

We used data from community treatment registers collated during a trial of four years MDA in schistosomiasis-endemic fishing communities of Lake Victoria, Uganda, to describe the proportion of eligible community residents who received anthelminthic treatment in this setting, to investigate reasons for non-receipt of treatment and predictors of receipt and of treatment refusal, and to investigate variability of treatment receipt across villages and whether this was associated with helminth prevalence.

## Methods

### Study setting and design

The LaVIISWA cluster randomised trial (ISRCTN47196031) was conducted in 26 fishing villages located in the Koome islands of Lake Victoria, Uganda. The Koome islands have a population of approximately 16,000 residents, with fishing and its related industries the main activities, and comprise the main island (Koome) and a number of smaller surrounding islands. The population of these islands is highly mobile, with much migration related to the movement of fish populations and to government policies related to these communities [18]. The islands are remote, being approximately 2–3 hours from the mainland by powered canoe. Prior to the commencement of LaVIISWA in 2013, the national programme of regular anthelminthic MDA was provided annually from 2004, although it was hampered by the cost of reaching these communities and by inadequate drug supply [19]; two rounds of treatment were missed, and for those that took place, treatment was mainly provided to the main island (where 10 of the study villages are situated) and not to the other islands (where the remaining 16 study villages are situated).

The LaVIISWA trial was originally designed to assess the impact of intensive versus standard anthelminthic treatment on allergy-related outcomes and helminth-related pathology [20]. Before the trial interventions began, we conducted a community-wide household survey (the LaVIISWA baseline survey) to collect baseline data on outcomes and other village- and individual-level characteristics. In this baseline survey (n = 2,316 participants across the 26 villages), 17% of participants reported receiving praziquantel treatment in the preceding 12 months and estimated *S*. *mansoni* infection prevalence was 52% based on Kato Katz analysis of a single stool sample [21]. Soil-transmitted helminths were also common, although less prevalent that *S*. *mansoni*: prevalence of hookworm (detected by PCR) was 22%, and prevalence of Trichuris (by Kato Katz) was 10%.

Following the LaVIISWA baseline survey, the 26 villages were randomised in a 1:1 ratio to receive either community-wide standard or community-wide intensive treatment for an initial period of three years. Standard treatment comprised annual single-dose praziquantel 40 mg/kg estimated by height pole to all individuals measuring ≥94cm (the standard height pole range) and twice-yearly single-dose albendazole 400mg to all individuals aged ≥1 year. Intensive treatment comprised quarterly single-dose praziquantel 40mg/kg using the extended height pole (≥60cm) to allow for treatment of young children, and quarterly triple-dose albendazole 400mg to all individuals aged ≥1 year. Pregnant and breastfeeding women were included in treatment distribution in both trial arms but in the intensive arm, they were given single-dose rather than triple-dose albendazole treatment. Standard treatment was also provided at schools in the communities.

Following three years of intervention, a further household survey of 3,350 residents across the 26 villages, was done to assess the impact of the interventions on the main trial outcomes. Results showed reductions in schistosomiasis prevalence and intensity compared to baseline, with prevalence based on Kato Katz of 39% in standard treatment villages and 23% in intensively-treated villages, and reductions in hookworm compared to baseline (prevalence based on PCR of 11% and 8% in standard and intensive treatment villages, respectively) but not Trichuris (prevalence based on Kato Katz of 10% and 8%, respectively); there was no effect of intensive versus standard intervention on allergy-related outcomes or on helminth-related pathology [22]. The intervention was then extended for a further year to assess its continued impact on parasitological outcomes and also to investigate effects on metabolic outcomes, with a final household survey conducted that included data on helminth infections from 2,066 residents across the 26 villages, with further reductions in prevalence of *S*. *mansoni* (prevalence based on Kato Katz of 34% in standard treatment villages and 21% in intensively-treated villages), and hookworm (prevalence based on PCR of 3% and 2%, respectively) but not in Trichuris (prevalence based on Kato Katz of 10% and 8% in standard and intensive treatment arms, respectively). Thus, data on albendazole and praziquantel treatment received over four years of anthelminthic intervention are available.

### Study procedures

Anthelminthic treatment was delivered in collaboration with the national and district Vector Control Programme and village health teams (VHTs); approximately three VHTs participated in each village. Substantial support was provided by the research team, with one to three field workers and two to five additional staff (such as clinical officers and nurses) participating in each village. Two members of Ministry of Health staff, one from the district Vector Control Programme and the other from the sub-county health centre, were also part of the treatment team. Treatment was delivered in each village over a two-day period, with advance notification given by VHTs. Each treatment round included multiple household visits and treatment provision for side effects. A household was defined as a group of people residing in a single physical structure, who slept in that building and who shared meals. As well as household visits, the study team set up a central base in each village, that people who were not found at home could attend for treatment, and also provided treatments in schools. With the exception of the second and third doses of albendazole in the intensive arm, all treatment was directly observed and drinking water was provided (juice for children) by the study team to ensure that participants were able to take treatments at the time they were provided. All team members were trained on treatment dispensing, including direct observation of treatment, to ensure consistency of distribution for all participants and across treatment rounds.

At baseline, residential households in each village were numbered sequentially with the numbers written in white paint to allow consistent identification of households for subsequent treatment rounds. A treatment register was established for each village, which listed all households and their known residents, together with their age and sex. Unique identifiers for each baseline resident were generated, comprised of a combination of village number, household number and person number (within that household). This register was updated at each treatment round, with household members checked and updated, and households in new buildings being allocated additional numbers. Thus at each treatment round, the register listed all community residents.

The treatment register was used at baseline and each subsequent round to record whether or not praziquantel and albendazole treatments were given, and the number of tablets dispensed to those who received treatment. For participants listed on the treatment register who did not receive the allocated treatments, the reasons for lack of treatment were recorded by study team members as ‘Absent (not for school)’ for participants who were not present (but not at school) on the days the treatment was delivered in a village, ‘Migrated’ when available information indicated the participant had moved to another address, ‘Refused’ when the participant was present but declined treatment, ‘At school’ when the participant was unavailable due to being at school, ‘Sick’ when the participant was unwell, or ‘Pregnant/breastfeeding’ if the participant was pregnant or breastfeeding and chose not to take treatment. Treatments given at schools were documented separately and were not included in this analysis due to difficulties in correctly identifying the household to which a child receiving treatment at school belonged, and to avoid “double counting” since the community-based registers aimed to collect information on treatment given by the study team regardless of where delivered.

### Ethical considerations

Ethical approval was given by the Uganda Virus Research Institute (reference number GC127), Uganda National Council for Science and Technology (reference number HS 1183) and London School of Hygiene & Tropical Medicine (reference number 6187). Before the trial intervention was started, community leaders provided written permission for their respective villages to receive MDA throughout the trial. Verbal consent was obtained from each community member before being offered the anthelminthic treatment. Individual written informed consent and assent was only requested from residents who participated in surveys conducted as part of the trial; for this written informed consent was received from all adults and emancipated minors and from parents or guardians for children; additional assent was obtained from children aged ≥8 years.

### Statistical analysis

The aims of this analysis were to quantify the proportion of eligible residents receiving anthelminthic treatment in this setting, to investigate reasons for non-receipt and predictors of receipt and of treatment refusal, and to investigate variability of treatment receipt across villages and whether this was associated with helminth prevalence. For this analysis, we define the proportion of eligible residents receiving treatment as the number who received treatment divided by the total number of eligible registered residents. Eligible registered residents were all residents aged ≥1 year for albendazole treatment and all residents aged ≥5 years for praziquantel treatment, following current WHO treatment guidelines. We define treatment refusal as documented refusal to receive a treatment (as described above participants could also not have received treatment due to absence from the household, being at school, being unwell, or because of pregnancy/breastfeeding), with the proportion of refusals calculated using all eligible registered residents as the denominator.

A post hoc power calculation indicated that this analysis had over 80% power to detect an absolute difference in mean treatment coverage between the two trial arms (each containing 13 villages) of 10–12%, assuming a standard deviation of 8–10%, and a 5% significance level.

Characteristics of baseline residents were summarised by trial arm. The mean number of residents registered at each treatment round was calculated. For each treatment round, praziquantel/albendazole receipt was calculated as the number of residents who received praziquantel/albendazole divided by the number of eligible residents listed in the village registers at that time point, based on the age criteria defined above and excluding those known to have migrated. Mean treatment receipt across treatment rounds was compared between trial arms using t-tests. Reasons for non-receipt of treatment were tabulated. Multinomial regression with linearised standard errors to allow for clustering at the village level was used to assess associations between resident socio-demographic characteristics and treatment receipt behaviour, categorised as received, did not receive due to absence, did not receive due to refusal, and did not receive due to other reasons (a merged category comprising absence due to school, illness, pregnancy or breastfeeding). Among participants who were resident in the same household for all treatment rounds during the four-year intervention period, mixed effects logistic regression was used to investigate whether sex and age were predictive of treatment receipt over time. In addition, the number and proportion who persistently refused treatment was tabulated, and age and sex examined as predictors of persistent non-receipt of treatment using logistic regression.

For each village, the mean proportion receiving praziquantel treatment among those eligible was calculated, averaging over all treatment rounds for that village over the 4 year period. These village-level mean proportions were then related to village-level prevalence of *S*. *mansoni* infection after four years of intervention, using linear regression, with an interaction term fitted to examine whether the relationship differed by trial arm. For each village, we also calculated the change *in S*. *mansoni* prevalence from the LaVIISWA baseline survey to the final, 4 year survey, and related this to the village-level mean proportion receiving praziquantel treatment over the 4 year period using linear regression and adjusting for baseline *S*. *mansoni* prevalence and trial arm. We conducted the same analysis for the association between change in Trichuris prevalence and mean proportion receiving albendazole treatment.

## Results

### Study participants

Between February and July 2013, 26 villages were enrolled in the trial and underwent their first treatment round, with 13 randomised to receive standard anthelminthic treatment and 13 randomised to receive intensive anthelminthic treatment. Treatment interventions were concluded four years later. For the 13 intensive treatment trial arm villages, data from 17 treatment rounds were available comprising the baseline treatment and 16 subsequent quarterly treatments. For the 13 standard treatment trial arm villages, data from a total of nine albendazole treatment rounds (baseline and eight twice-yearly treatments) and from a total of five praziquantel treatments (baseline and four annual treatments) were available.

**Fig 1:**
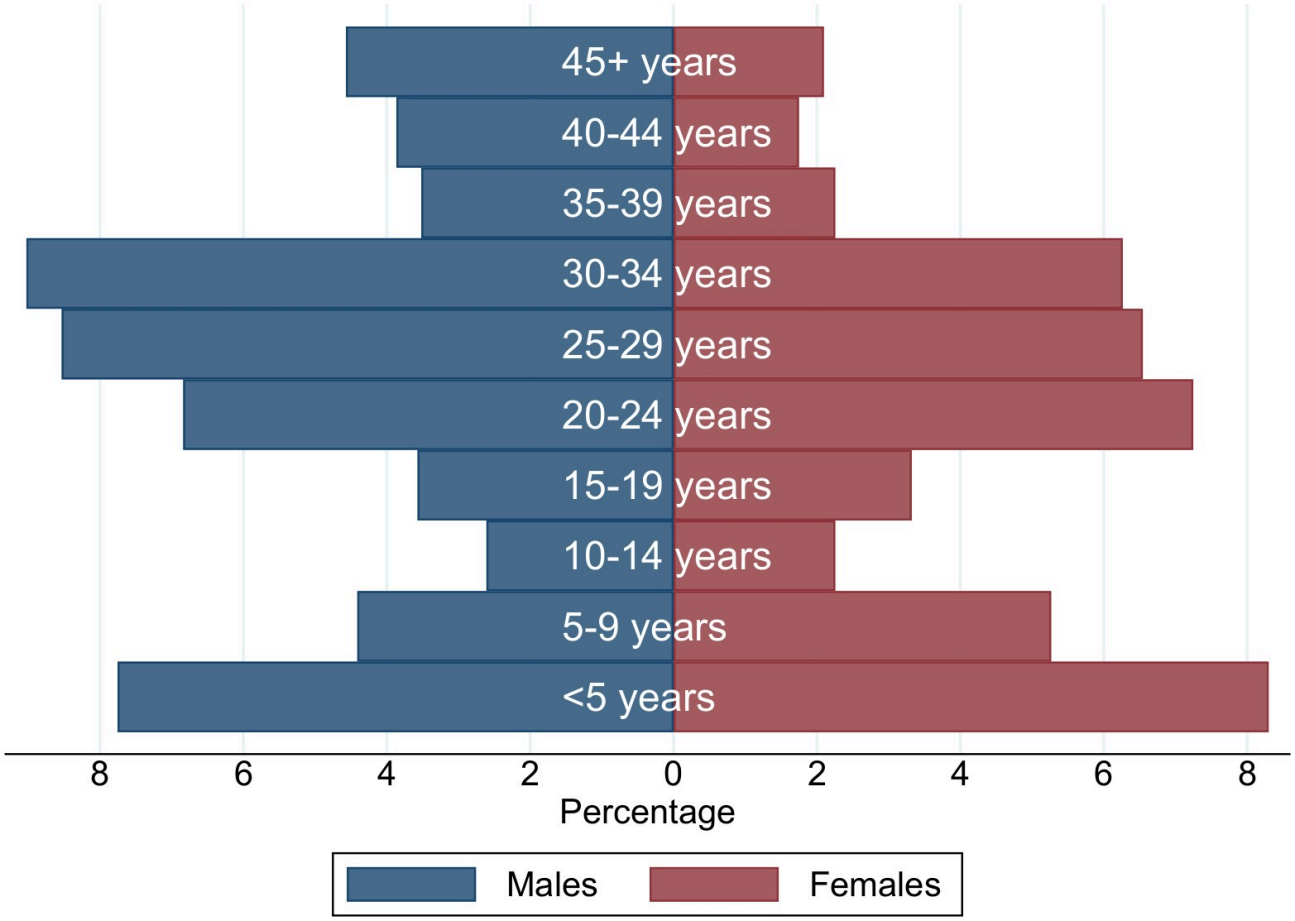
Age-sex distribution of village residents at baseline.

At baseline, 13,966 residents were listed in village registers (7,498 standard treatment arm, 6,468 intensive treatment arm). Of these 13,097 were aged ≥1 year and were eligible for albendazole treatment, and 11,095 were aged ≥5 years and were eligible for praziquantel treatment. The age-sex distribution of baseline residents is shown in Fig 1. The age distribution was bi-modal; children aged 5–19 years were underrepresented due to many attending school on the mainland (although there are some schools on the islands). Among adults, there were more men than women, especially among the older age groups, reflecting the fact that fishing and its related activities are the main occupations in this setting.

Residents of the study setting were highly mobile, moving both within and between villages, and/or migrating to or from the study area entirely. Thus, longitudinal follow-up of individuals was challenging. In total, across all 26 villages, there were 49,457 residents recorded on registers at any point during the four-year trial intervention period (23,298 in the standard intervention villages, and 26,159 in the intensive intervention villages). By comparison, the mean total population of the 26 villages during the 17 treatment rounds was 13,382 (7,153 in the standard intervention villages, and 6,229 in the intensive intervention villages). From interrogating names of participants, it was clear that many moved dwelling multiple times during the study period, but since registers included household number in the identifier for individual participants, it was not possible to track individual treatment behaviour unless the participant had remained in the same household throughout the study period. We estimate that a total of 1,466 residents (882 standard intervention arm, 584 intensive intervention arm) were present in the same household for all treatment rounds throughout the full four-year intervention period.

### The proportion receiving treatment and its predictors

**Fig 2:**
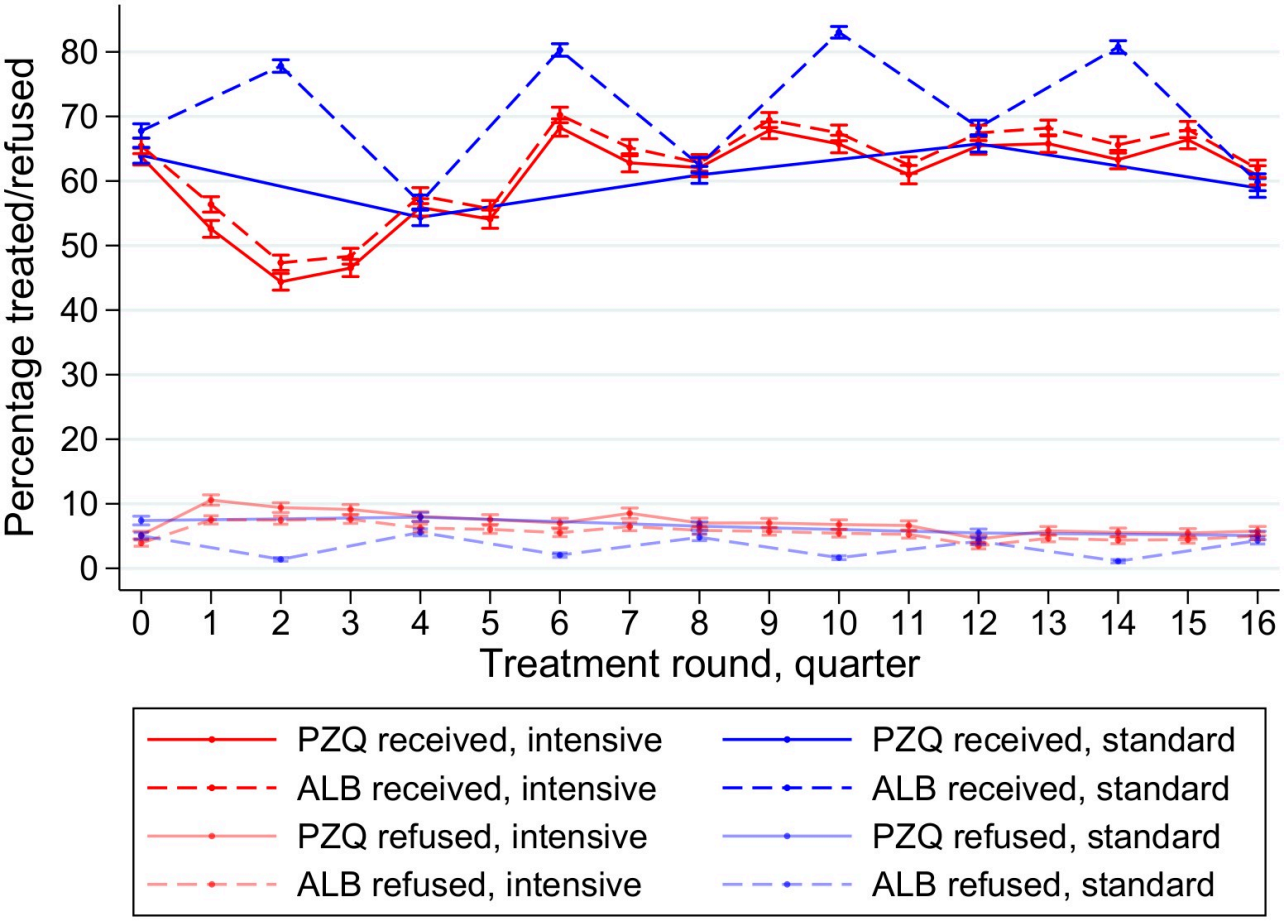
Proportion of eligible residents receiving treatment and proportion of eligible residents refusing treatment over four years of anthelminthic treatment, by trial arm and drug.

There was a general trend of an increase in proportion of eligible residents receiving treatment over time, for both trial arms, and for both albendazole and praziquantel treatments (Fig 2). Averaging over all treatment rounds, the proportion of eligible residents receiving praziquantel treatment was lower than for albendazole (60% versus 65%), particularly in the standard arm (praziquantel 61%, albendazole 71%) compared to the intensive arm (praziquantel 60%, albendazole 62%). In the standard arm, the proportion receiving albendazole treatment was lower in the five treatment rounds at which praziquantel treatment was also given (concurrently) compared to the four treatment rounds at which praziquantel treatment was not given (63.0% versus 80.5%, mean difference = 17.5%, 95% CI: 11.0–23.9%, p = 0.004). In the intensive arm, albendazole treatment and praziquantel treatment were given concurrently at all rounds.

**Fig 3:**
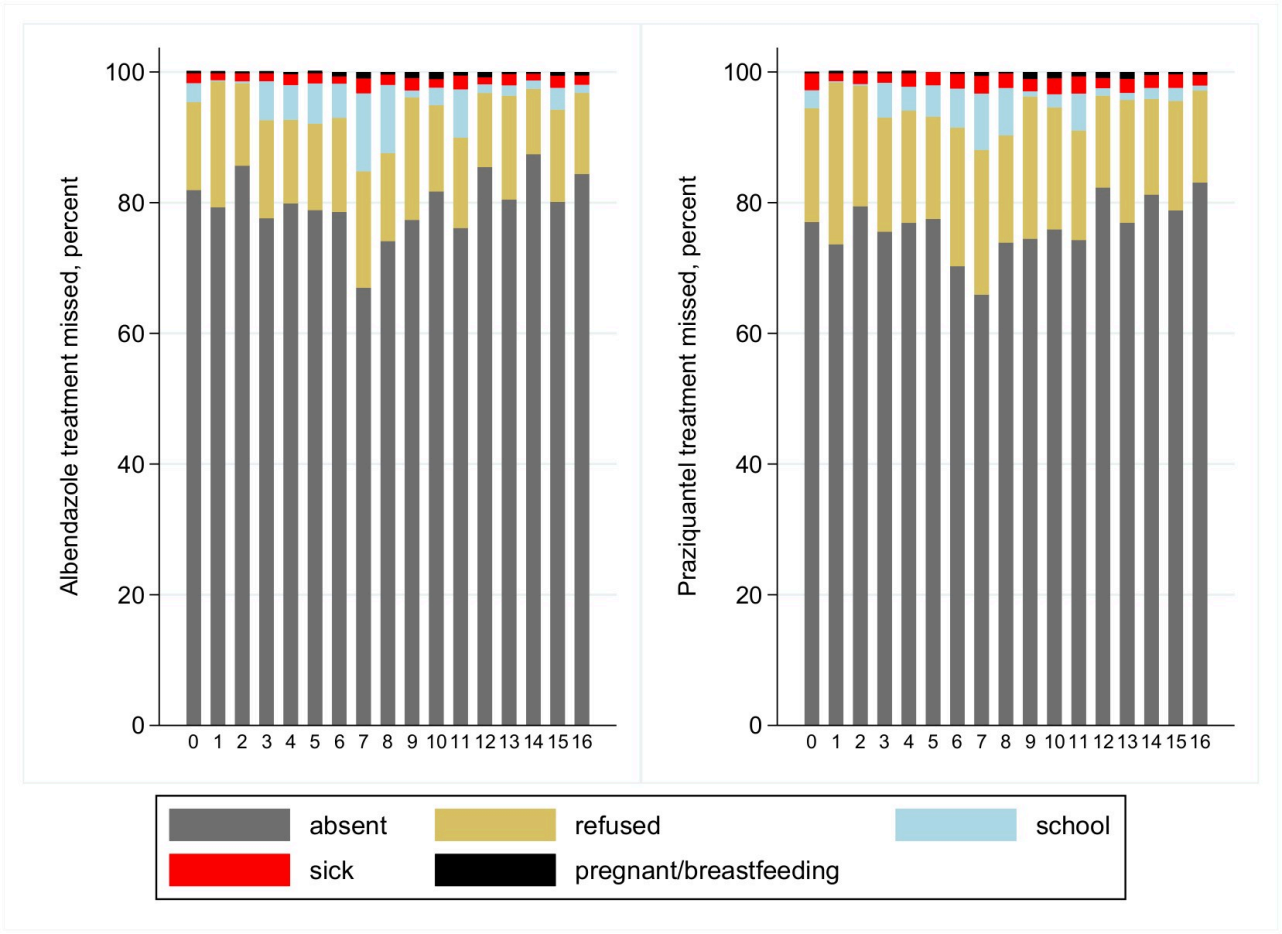
Reasons for non-receipt of albendazole and praziquantel treatment, by treatment round.

By far the most common reason for an eligible resident to not receive a scheduled treatment was (non-permanent) absence from the home during the week that the team were providing treatment in that village (Table 1). The next most common reason was refusal, and this was higher for praziquantel than it was for albendazole (17.6% versus 13.8% among those who were eligible but did not take treatment, p<0.001, Fig 2). Recorded reasons for non-receipt of either albendazole or praziquantel treatment were consistent over time (Fig 3).

**Table 1:** Reasons for non-receipt of anthelminthic treatment.

	Albendazole			Praziquantel		
Reason	Standard	Intensive	Overall	Standard	Intensive	Overall
**Absent (not for school)**	84.6%	79.1%	**80.8%**	78.5%	76.7%	**77.2%**
**Refused**	11.5%	14.9%	**13.8%**	16.8%	17.9%	**17.6%**
**At school**	2.5%	4.0%	**3.5%**	2.2%	3.0%	**2.8%**
**Sick**	0.9%	1.7%	**1.5%**	2.1%	2.0%	**2.0%**
**Pregnant/breastfeeding**	0.5%	0.3%	**0.4%**	0.5%	0.4%	**0.4%**

For both albendazole and praziquantel, the proportion taking treatment was similar for both males and females (Tables 2 and 3). However, females were less likely to not receive treatment due to absence, but more likely to not receive treatment due to refusal and for other reasons (specifically, pregnancy and breastfeeding). For both drugs, the proportion of residents taking treatment differed by age, being lowest in 10–19 years olds (Table 3). Children were most likely not to receive treatment due to absence and least likely not to receive treatment due to refusal, while older adults were most likely to miss treatment due to illness.

**Table 2:** Age and sex as predictors of albendazole receipt behaviour.

	Received	Did not receive due to absence		Did not receive due to refusal		Did not receive due to other reasons^1^	
Predictor	%	%	RRR (95% CI)^1^	%	RRR (95% CI)	%	RRR (95% CI)
**Sex**							
Male	65%	29%	1 (ref)	4.4%	1 (ref)	1.4%	1 (ref)
Female	65%	27%	0.93 (0.87, 0.99)	5.2%	1.28 (1.12, 1.45)	2.4%	1.54 (1.29, 1.85)
**Age group (years)**							
<10	66%	28%	1.00 (0.89, 1.11)	1.7%	0.28 (0.23, 0.33)	3.9%	3.93 (2.18, 7.08)
10–19	60%	34%	1.30 (1.15, 1.46)	3.7%	0.65 (0.57, 0.74)	2.5%	2.76 (1.88, 4.06)
20–29	65%	28%	1 (ref)	6.1%	1 (ref)	1.0%	1 (ref)
30–39	66%	26%	0.92 (0.87, 0.97)	6.9%	1.13 (1.00, 1.26)	0.7%	0.72 (0.59, 0.89)
40–49	68%	26%	0.88 (0.79, 0.98)	5.7%	0.92 (0.80, 1.07)	0.6%	0.66 (0.42, 1.03)
50+	67%	26%	0.89 (0.78, 1.01)	6.2%	1.05 (0.88, 1.26)	1.6%	1.83 (1.22, 2.73)

^1^Other reasons: any of absence due to school, illness, pregnancy or breastfeeding;

^2^RRR: Relative risk ratio from multinomial regression;

^3^CI: confidence interval

**Table 3:** Age and sex as predictors of praziquantel receipt behaviour.

	Received	Did not receive due to absence		Did not receive due to refusal		Did not receive due to other reasons^1^	
Predictor	%	%	RRR (95% CI)^2^	%	RRR (95% CI)	%	RRR (95% CI)
**Sex**							
Male	61%	32%	1 (ref)	5.9%	1 (ref)	1.5%	1 (ref)
Female	60%	29%	0.90 (0.83, 0.98)	8.2%	1.45 (1.29, 1.63)	2.8%	1.76 (1.42, 2.17)
**Age group (years)**							
<10	56%	35%	1.24 (1.06, 1.46)	2.1%	0.29 (0.22, 0.37)	6.9%	6.28 (3.33, 11.82)
10–19	56%	36%	1.30 (1.14, 1.48)	4.9%	0.67 (0.59, 0.76)	3.0%	2.75 (1.90, 3.99)
20–29	61%	30%	1 (ref)	8.0%	1 (ref)	1.2%	1 (ref)
30–39	62%	28%	0.92 (0.86, 0.98)	8.8%	1.11 (1.00, 1.23)	1.0%	0.86 (0.72, 1.03)
40–49	64%	28%	0.88 (0.78, 0.98)	7.6%	0.97 (0.85, 1.10)	0.9%	0.76 (0.48, 1.22)
50+	61%	28%	0.89 (0.78, 1.02)	8.3%	1.13 (0.95, 1.33)	2.8%	2.64 (1.67, 4.18)

^1^Other reasons: any of absence due to school, illness, pregnancy or breastfeeding;

^2^RRR: Relative risk ratio from multinomial regression;

^3^CI: confidence interval

We examined age and sex as predictors of treatment receipt among the 1,466 participants who were present in the same household for all treatment rounds throughout the full four-year intervention period, using mixed effects models. Sex was not associated with receipt of either albendazole or praziquantel (Table 4). There were some patterns of association for age, with 10–19 year olds being least likely to receive treatment, although this does not take into account that they may have been treated at school.

**Table 4:** Longitudinal analysis of age and sex as predictors of treatment receipt behaviour.

	Albendazole			Praziquantel		
Predictor	Receipt^1^	aOR (95% CI)	p-value	Receipt^1^	aOR (95% CI)	p-value
**Sex**						
Male	73.4%	1 (ref)		69.5%	1 (ref)	
Female	74.3%	1.07 (0.93, 1.24)	0.32	69.4%	1.02 (0.86, 1.21)	0.82
**Age group (years)**						
<10	72.7%	0.90 (0.72, 1.12)	0.001	69.0%	0.91 (0.66, 1.25)	0.02
10–19	65.9%	0.61 (0.44, 0.84)		62.4%	0.63 (0.43, 0.91)	
20–29	73.3%	1 (ref)		68.7%	1 (ref)	
30–39	75.0%	1.10 (0.91, 1.34)		70.8%	1.12 (0.90, 1.40)	
40–49	76.2%	1.22 (0.98, 1.53)		70.9%	1.17 (0.91, 1.50)	
50+	72.6%	0.99 (0.70, 1.40)		67.3%	0.93 (0.63, 1.37)	

^1^Receipt calculated as number taking treatment divided by number registered; aOR: adjusted odds ratio, CI: confidence interval

Only forty-two participants (2.9%) received no praziquantel treatment over the full 4-year period. There were no clear differences in age and sex distributions between participants who never received praziquantel and those who received at least one dose (Supplementary Table). For albendazole, only 6 participants (0.4%) never received treatment. Socio-demographic predictors of persistent non-receipt of albendazole were not examined due to this small number.

### Variability of treatment receipt across villages and relationship with helminth prevalence

There was wide variability in proportions receiving treatment across the villages that took part in the trial (S1 and S2 Figs). Averaging over treatment rounds, the mean proportion receiving albendazole treatment per village ranged from 50.2% to 85.2% overall (60.7%-85.2% standard arm, 50.3%-84.3% intensive arm). Mean praziquantel receipt ranged from 48.3% to 83.8% overall (49.2%-74.5% standard arm, 48.3%-83.8% intensive arm). A sustained intervention coverage of >75% (WHO target) was achieved in two villages. There was some suggestion of an inverse relationship between the proportion receiving praziquantel treatment and schistosomiasis prevalence at the end of the four-year trial in the standard arm villages (linear regression coefficient: -0.94 (95% CI: -2.06, 0.17), p = 0.09), but not in the intensive arm villages (linear regression coefficient -0.01 (95% CI: -0.83, 0.82), p = 0.99, p-value for interaction 0.15). However, further analysis using village-level change in *S*. *mansoni* prevalence from baseline to 4 years as the outcome found only weak evidence of association with the village-level proportion receiving praziquantel treatment over the 4-year period (linear regression coefficient -0.29 (95% CI: -0.74, 0.16), p = 0.17, adjusted for baseline prevalence and trial arm). Baseline *S*. *mansoni* prevalence was highly correlated with 4-year *S*. *mansoni* prevalence, both overall (correlation coefficient 0.77) and in both standard and intensive treatment arms (correlation coefficients 0.80 and 0.78, respectively), and there was a greater reduction in *S*. *mansoni* prevalence in those villages where prevalence was highest at baseline, regardless of praziquantel uptake or intensive versus standard intervention (adjusted linear regression coefficient -0.30 (-0.54, -0.06), p = 0.02). A similar analysis of the association between mean albendazole treatment coverage and change in Trichuris infection from baseline to 4-years found no association (adjusted linear regression coefficient -0.04, (-0.40, 0.31), p = 0.80).

## Discussion

In these schistosomiasis-endemic fishing villages, mean praziquantel and albendazole treatment coverage was 60% and 65%, respectively. Absence was by far the most frequently recorded reason for missing a MDA treatment round, with refusal only reported for around one in seven of those not receiving treatment in any given treatment round. Longitudinal analysis of a subgroup of residents who did not move during the study period found that persistent non-receipt of treatment in this subgroup was rare. The proportion receiving our community-based MDA intervention was lowest among school-aged children, with refusal more common among females and older participants. There was wide variability in proportions receiving treatment across villages, with few achieving WHO target coverage.

WHO targets praziquantel treatment coverage of 75% in schistosomiasis-endemic areas [6]. Despite extensive efforts to achieve high treatment coverage, including community engagement activities throughout the study, advance notice of visits to villages for treatment provision; two-day stays by treatment teams in communities; repeated household visits; substantial support by the research team including field workers, clinical officers and nurses; treatment provision for adverse events; and provision of drinking water (and juice for children), only two of our 26 villages achieved a mean praziquantel treatment coverage above this level. However, even in these villages, which both received the intensive treatment intervention, 25–30% of village residents remained infected with *S*. *mansoni* based on Kato Katz analysis of a single stool sample after four years of quarterly praziquantel treatment with high coverage. In schistosomiasis high-risk communities such as this, MDA alone, even offered very frequently, is insufficient for the control of schistosomiasis. Additional interventions, such as WASH, vector control, or an effective vaccine, are required.

As might be expected, coverage estimates in this resource-intensive non-programmatic trial, compare favourably with some previous reports from pragmatic trials or programmatic studies [23]. However, it is important to note that our findings are unlikely to be comparable with those from a routine MDA programme, where treatment is provided by VHTs alone (rather than working as part of a trial team as was the case here) and is generally available in the community over an extended period of time [24].

There was a suggestion of an inverse relationship between the proportion of village residents receiving praziquantel treatment and reduction in schistosomiasis prevalence. However, trial arm (intensive versus standard) and higher baseline prevalence were more important predictors of reduction in prevalence over the four years. These findings should be treated with caution since they are based on 26 data points (the number of trial clusters) and other community-level factors such as proximity to water, availability of WASH, and socio-economic factors are likely to confound the relationship between treatment receipt and prevalence.

Absence during treatment rounds was by far the most common reason for not receiving MDA. This is consistent with findings from Mayuge District in Uganda [16], where non-receipt of treatment was more likely to be due to not being offered treatment rather than systematic non-compliance. In our trial, every effort was made to advertise the forthcoming treatment and offer multiple opportunities of receiving it, but despite this, coverage remained sub-optimal. This may have been partly due to the fact that treatment rounds took place over a two-day period in each village, thus temporary absences would have had a significant impact; a longer treatment period may have been beneficial, although treatment of the whole community in a short period is likely to have the best effect on transmission. In the standard treatment arm, we found that the proportion taking albendazole treatment decreased somewhat when praziquantel was offered concurrently, compared to treatment rounds where albendazole only was offered, suggesting that a small proportion of the absence could actually be due to avoidance. This, taken together with the higher refusal rates for praziquantel, and the fact that, among participants for whom longitudinal data were available, a somewhat higher proportion missed all praziquantel treatments than missed all albendazole treatments, provides some support to the perception that praziquantel treatment is less acceptable than albendazole treatment [25]. This is probably due to the abdominal discomfort and other adverse effects that may be induced by praziquantel treatment, particularly for heavy infections. Women were more likely to refuse praziquantel treatment than men. Reasons for this are unclear, but could be due to concerns about taking during pregnancy or a belief that they are less likely to have schistosomiasis due to less frequent or obvious lake contact [26]. Our results contrast with studies from other settings that have found little difference in treatment receipt by age and, or gender [16, 17], instead finding that factors such as socio-economic status and sanitation had greater predictive utility; unfortunately we did not collect information on these variables on the treatment registers in our study so could not investigate them further. Further qualitative work is needed to provide insight and understanding of these patterns. However, the reasons for missing treatment in our study (predominantly absence) were often similar to those reported by other studies.

The lowest proportion receiving albendazole treatment was in the 10–19 years age group. This could be partially explained by absence for school attendance. We used a combined delivery strategy of both school-based and community-wide provision of MDA, as recommended by a recent systematic review to optimise praziquantel coverage [27]. However, a limitation of our study was that treatments given at schools were documented separately and not combined with our community treatment registers due to difficulties in identifying children’s households. Thus, we are likely to be underestimating the coverage in school-aged children because our analysis was focussed on community-based registers.

Unfortunately, due to the highly mobile population in this study setting, we were unable to trace many individuals for the entire four-year study period. Thus longitudinal analyses to assess individual time trends in treatment receipt and/or individual level characteristics that might influence persistent non-receipt or refusal of treatment were restricted to around 1,500 participants. Reassuringly, analysis of this “non-mobile” subgroup suggested that very few individuals persistently missed every single treatment round, whether due to absence or refusal. Studies in neighbouring districts in Uganda have found a similarly low level of systematic noncompliance [16, 28]. This suggests that further repeated visits over an extended period may have utility for increasing coverage, although it is important to note that this “non-mobile” subgroup may not be representative of all residents of these fishing communities. The degree of mobility in these fishing communities causes many challenges for the coverage and for estimating the coverage of MDA [19]. A further limitation was the small number of covariates available for village residents, thus we were not able to assess socio-demographic determinants of treatment receipt beyond age and sex, and thus our findings in relation to these may be confounded by unmeasured variables, such as WASH measures and socio-economic status. This lack of other covariates is a reflection of the fact that assessment of factors associated with treatment receipt was not the primary aim of the LaVIISWA study.

A further limitation of the study is that the second and third doses of albendazole in the intensive trial arm were not directly observed. Therefore, for intensive arm albendazole treatment, we can only accurately assess the proportion receiving the first dose. However, all other treatments were directly observed and thus reduced compliance (where treatments are provided to an individual but not taken) is unlikely to have been a factor in our study [29]. A further strength is that data on treatment receipt were objectively collected by the research team using registers of residents, and not based on self-report, although previous studies have suggested that individuals are able to recall previous treatment receipt with reasonable accuracy [30].

In summary, our findings demonstrate that in schistosomiasis high-risk communities such as the fishing communities of Lake Victoria, MDA with praziquantel is unlikely to be sufficient to achieve control. A combination of community-wide and school-based treatment strategies and educational interventions may be required to achieve WHO target MDA coverage [27]. Furthermore, an effective vaccine [31] and additional measures targeting the full *S*. *mansoni* life cycle, notably improved provisions for water supply and sanitation [32], are needed to move towards elimination.

## Supporting information

S1 Fig Village-level mean proportion of eligible residents receiving albendazole treatment, by trial arm. (TIF)

S2 Fig Village-level mean proportion of eligible residents receiving praziquantel treatment, by trial arm. (TIF)

S1 Table Predictors of persistent praziquantel non-treatment among eligible residents who remained in the same household throughout the intervention period. (DOCX)
