# Supplementary figures and images for: Anthelminthic treatment receipt and its predictors in Lake Victoria fishing communities, Uganda: Intervention coverage results from the LaVIISWA cluster randomised trial
Source: PLoS Negl Trop Dis. 2020 Oct 19;14(10):e0008718. doi: 10.1371/journal.pntd.0008718 (PMC7595614; doi:10.1371/journal.pntd.0008718)

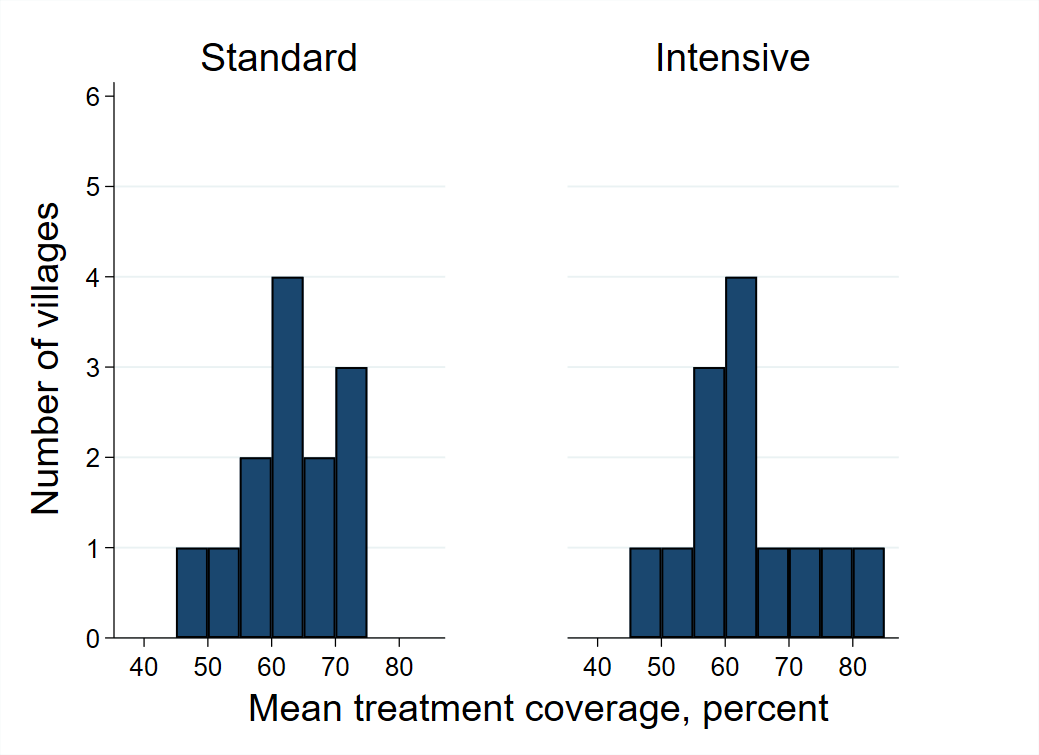

Supplement: S1 Fig — (TIF) [file pntd.0008718.s001.tif]

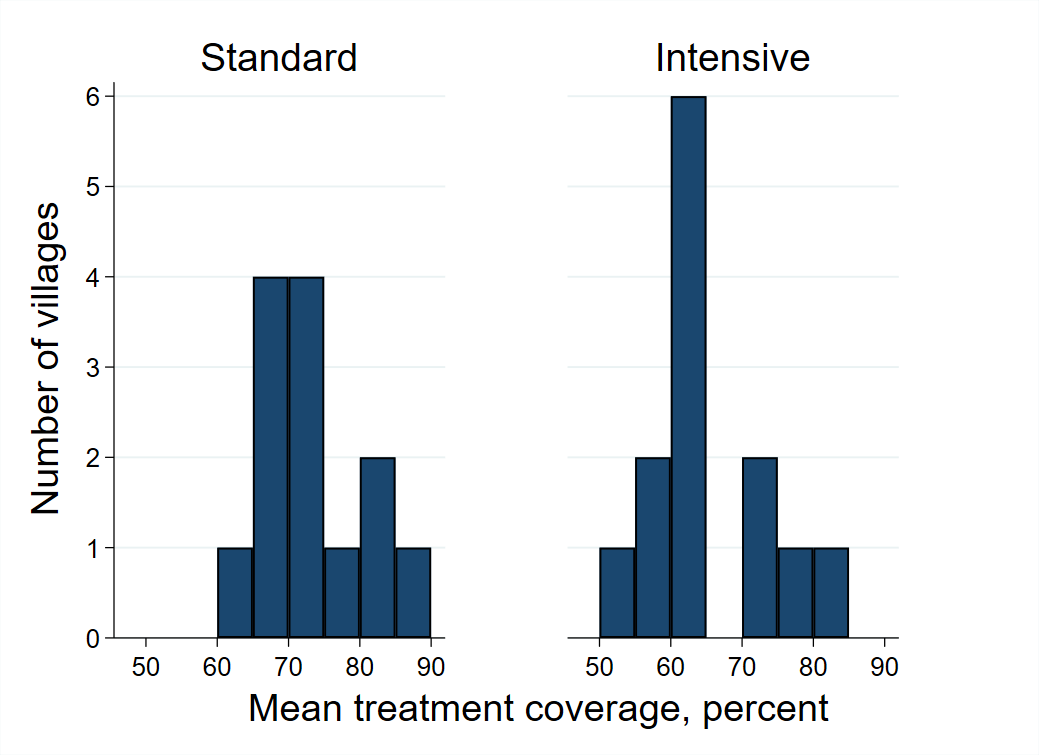

Supplement: S2 Fig — (TIF) [file pntd.0008718.s002.tif]
