# Supplementary material for: Anthelminthic treatment receipt and its predictors in Lake Victoria fishing communities, Uganda: Intervention coverage results from the LaVIISWA cluster randomised trial
Source: PLoS Negl Trop Dis. 2020 Oct 19;14(10):e0008718. doi: 10.1371/journal.pntd.0008718 (PMC7595614; doi:10.1371/journal.pntd.0008718)
Supplement: S1 Table — (DOCX) [file pntd.0008718.s003.docx]

**Supplementary Table. Predictors of persistent praziquantel non-treatment among eligible residents who remained in the same household throughout the intervention period**

| **Predictor** | **% never treated** | **Adjusted OR (95% CI)** | **p-value** |
| --- | --- | --- | --- |
| **Sex** |  |  |  |
| Male | 3.0% | 1 |  |
| Female | 2.8% | 0.97 (0.50, 1.88) | 0.92 |
| **Age group (years)** |  |  |  |
| <10 | 3.3% | 1.17 (0.33, 4.20) | 0.47 |
| 10-19 | 3.9% | 1.38 (0.72, 2.63)) |  |
| 20-29 | 2.8% | 1 |  |
| 30-39 | 2.8% | 0.99 (0.41, 2.37) |  |
| 40-49 | 1.5% | 0.53 (0.15, 1.93) |  |
| 50+ | 7.0% | 2.59 (0.85, 7.87) |  |
